# The Local CNP/GC-B system in growth plate is responsible for physiological endochondral bone growth

**DOI:** 10.1038/srep10554

**Published:** 2015-05-27

**Authors:** Kazumasa Nakao, Kenji Osawa, Akihiro Yasoda, Shigeki Yamanaka, Toshihito Fujii, Eri Kondo, Noriaki Koyama, Naotetsu Kanamoto, Masako Miura, Koichiro Kuwahara, Haruhiko Akiyama, Kazuhisa Bessho, Kazuwa Nakao

**Affiliations:** 1 Department of Oral and Maxillofacial Surgery, Kyoto University Graduate School of Medicine, 54 Kawahara-cho, Shogo-in, Sakyo-ku, Kyoto 606-8507, Japan; 2 Department of Diabetes, Endocrinology and Nutrition, Kyoto University Graduate School of Medicine, 54 Kawahara-cho, Shogo-in, Sakyo-ku, Kyoto 606-8507, Japan; 3 Department of Cardiovascular Medicine, Kyoto University Graduate School of Medicine, 54 Kawahara-cho, Shogo-in, Sakyo-ku, Kyoto 606-8507, Japan; 4 Department of Orthopaedic Surgery, Gifu University Graduate School of Medicine, 1-1 Yanagido, Gifu 501-1194, Japan; 5 Medical Innovation Center, Kyoto University Graduate School of Medicine, 53 Shogoin-Kawahara-cho, Sakyo-ku, Kyoto 606-8507, Japan

## Abstract

Recent studies revealed C-type natriuretic peptide (CNP) and its receptor, guanylyl cyclase-B (GC-B) are potent stimulators of endochondral bone growth. As they exist ubiquitously in body, we investigated the physiological role of the local CNP/GC-B in the growth plate on bone growth using cartilage-specific knockout mice. Bones were severely shorter in cartilage-specific CNP or GC-B knockout mice and the extent was almost the same as that in respective systemic knockout mice. Cartilage-specific GC-B knockout mice were shorter than cartilage-specific CNP knockout mice. Hypertrophic chondrocyte layer of the growth plate was drastically reduced and proliferative chondrocyte layer, along with the proliferation of chondrocytes there, was moderately reduced in either cartilage-specific knockout mice. The survival rate of cartilage-specific CNP knockout mice was comparable to that of systemic CNP knockout mice. The local CNP/GC-B system in growth plate is responsible for physiological endochondral bone growth and might further affect mortality via unknown mechanisms.

The natriuretic peptide family consists of three structurally related endogenous ligands: atrial natriuretic peptide (ANP), brain natriuretic peptide (BNP), and C-type natriuretic peptide (CNP)^1^. The three natriuretic peptide receptors are membrane-bound proteins, two of which are biologically active guanylyl cyclase (GC)-coupled receptors (GC-A and GC-B), and one of which is a biologically silent metabolic clearance receptor (C-receptor)^2^. ANP and BNP, which exert their biological activities through GC-A^3^, are cardiac hormones produced predominantly in the atrium and ventricle of the heart, respectively^1^; these hormones are implicated in the regulation of blood pressure, water, and electrolyte balance^4^. On the other hand, CNP exerts its biological activities through GC-B, and is present in various tissues including brain, pituitary, blood vessels, ovary, testis, and cartilage^5^,^6^,^7^,^8^,^9^,^10^,^11^,^12^,^13^,^14^,^15^,^16^,^17^,^18^.

Most bones in mammals, including vertebrae and the long bones of limbs, are formed through endochondral ossification, which involves the conversion of an initial cartilage template into bone via proliferation, hypertrophy, cell death, and osteoblastic replacement in the growth plate. In previous studies, we and another group showed that mice systemically depleted of CNP or GC-B exhibit severely impaired growth of vertebrae and long bones^16^,^17^. Given that both CNP and GC-B are expressed in the growth plate cartilage of vertebrae and long bones^16^, it is possible that the local CNP/GC-B system in the growth plate contributes in some way to physiological endochondral bone growth. However, the relevance of the contribution to physiological bone growth by CNP, either in the growth plate or originating in other tissues, is still elusive; direct or indirect effects of CNP from various tissues other than cartilage, including brain that is regarded to contain the most abundant CNP in body and pituitary, the center of the growth hormone-insulin like growth factor-I (GH/IGF-I) axis that regulates endochondral bone growth^19^, might play any roles on physiological endochondral bone growth. Actually, we developed transgenic mice with an elevated plasma concentration of CNP under the control of human serum amyloid P component promoter and exhibited that these mice showed prominent skeletal overgrowth phenotype, indicating that CNP can humorally affect endochondral bone growth^20^. Furthermore, precise manners of the stimulation of endochondral ossification of the CNP/GC-B system in the growth plate are still elusive. Therefore, in the present study, we generated cartilage-specific CNP knockout mice using the Cre recombinase (Cre)-loxP system, which enables targeted depletion of an intended gene, and used these mice to investigate the physiological effects of CNP on endochondral bone growth in the growth plate. Furthermore, we knocked down GC-B in the growth plate in mice using the same Cre-loxP system, and further investigated the physiological roles of the CNP/GC-B system on endochondral bone growth there.

## Results

### Generation and gross phenotypes of mice with cartilage-specific deletion of CNP

**Figure 1:**
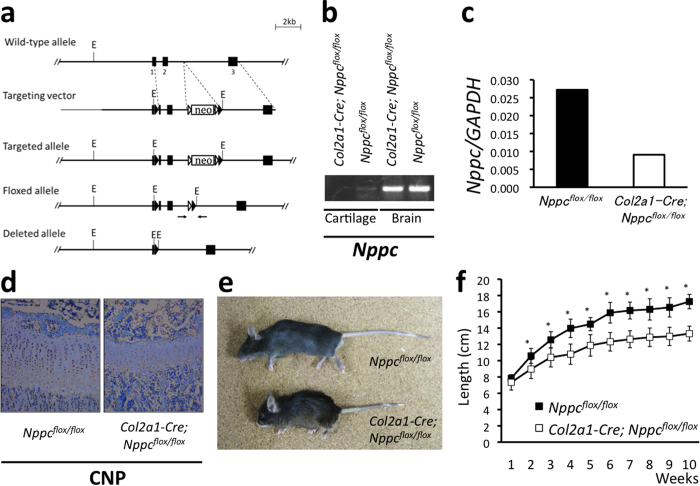
Generation and gross appearance of *Col2a1-Cre; Nppc*^*flox/flox*^ mice. (**a**) Conditional targeting of *Nppc*. Restriction enzymes: E, EcoRI; Arrows: PCR primers. Triangles (black): loxP sites. Triangles (white): FRT sites. (**b**) RT-PCR analysis of *Nppc* in cartilage and brain of *Col2a1-Cre; Nppc*^*flox/flox*^ and *Nppc*^*flox/flox*^ mice. The full-length gel is presented in Supplementary Figure S1. (**c**) Quantitative real-time RT-PCR analysis for CNP expression in *Nppc*^*flox/flox*^ and *Col2a1-Cre; Nppc*^*flox/flox*^ mice. **(d)** Immunohistochemical analysis of CNP in growth plates of *Nppc*^*flox/flox*^ and *Col2a1-Cre; Nppc*^*flox/flox*^ mice. (**e**) Gross appearance of *Nppc*^*flox/flox*^ (upper) and *Col2a1-Cre; Nppc*^*flox/flox*^ (lower) mice. (**f**) Growth curves of naso-tail lengths of *Nppc*^*flox/flox*^ (■) and *Col2a1-Cre; Nppc*^*flox/flox*^ (□) mice. *: P < 0.01.

In order to inactivate the murine CNP gene (*Nppc*) in the chondrogenic cell lineage, we used mice carrying *Nppc*^*flox*^, a *Nppc* allele in which the DNA segment that includes the 3’ part of exon 1 and all of exon 2 is flanked by *loxP* sites; the floxed portions of the exons encode the entire structure of CNP (Fig. 1a). Both heterozygous and homozygous mice carrying *Nppc*^*flox*^ were viable and fertile, and they exhibited no noticeable phenotypic changes. We crossed mice heterozygous for the *Nppc*^*flox*^ allele with mice heterozygous for the *Col2a1–Cre* transgene, which enables targeted expression of the Cre recombinase in cartilage^21^. Offspring that inherited both the *Nppc*^*flox*^ allele and the *Col2a1–Cre* transgene were mated with mice inheriting only the *Nppc*^*flox*^ allele, in order to yield homozygous *Nppc*^*flox*^ mice expressing Cre recombinase specifically in cartilage (*Col2a1–Cre; Nppc*^*flox/flox*^). Cartilage-specific deletion of CNP expression in the resultant *Col2a1–Cre; Nppc*^*flox/flox*^ mouse was confirmed by RT-PCR (Fig. 1b and Supplementary Fig. S1). Quantitative real-time RT-PCR analyses revealed that the expression of CNP was dramatically decreased in the cartilage of *Col2a1–Cre; Nppc*^*flox/flox*^ mouse compared to that of *Nppc*^*flox/flox*^ mouse as the control (Fig. 1c). The decrease of CNP protein in the growth plate of *Col2a1–Cre; Nppc*^*flox/flox*^ mouse was exhibited by the immunohistochemical staining using anti-CNP antibody (Fig. 1d).

Figure 1e depicts the gross appearance of a *Col2a1–Cre; Nppc*^*flox/flox*^ mouse compared to that of an *Nppc*^*flox/flox*^ mouse at the age of 10 weeks. At this age, the *Col2a1–Cre; Nppc*^*flox/flox*^ mouse was shorter than the *Nppc*^*flox/flox*^ mouse. The growth curves reveal that at the first week after birth, the *Col2a1–Cre; Nppc*^*flox/flox*^ mice were only slightly smaller than the *Nppc*^*flox/flox*^ mice; naso-anal and naso-tail lengths of *Col2a1–Cre; Nppc*^*flox/flox*^ mice were 92.0% and 93.6%, respectively, of the corresponding lengths in the controls (Fig. 1f and Supplementary Fig. S2). Later, the growth of *Col2a1–Cre; Nppc*^*flox/flox*^ mice was greatly retarded relative to controls: at the age of 6 weeks, naso-anal and naso-tail lengths of *Col2a1–Cre; Nppc*^*flox/flox*^ mice were 78.6% and 77.6%, respectively, of the control lengths. After 6 weeks, *Col2a1–Cre; Nppc*^*flox/flox*^ mice grew similarly to control mice, and at 10 weeks the naso-anal and naso-tail lengths of *Col2a1–Cre; Nppc*^*flox/flox*^ mice were 78.6% and 75.6%, respectively, of the control lengths (Fig. 1f and Supplementary Fig. S2).

### Skeletal phenotypes of cartilage-specific CNP knockout mice

**Figure 2:**
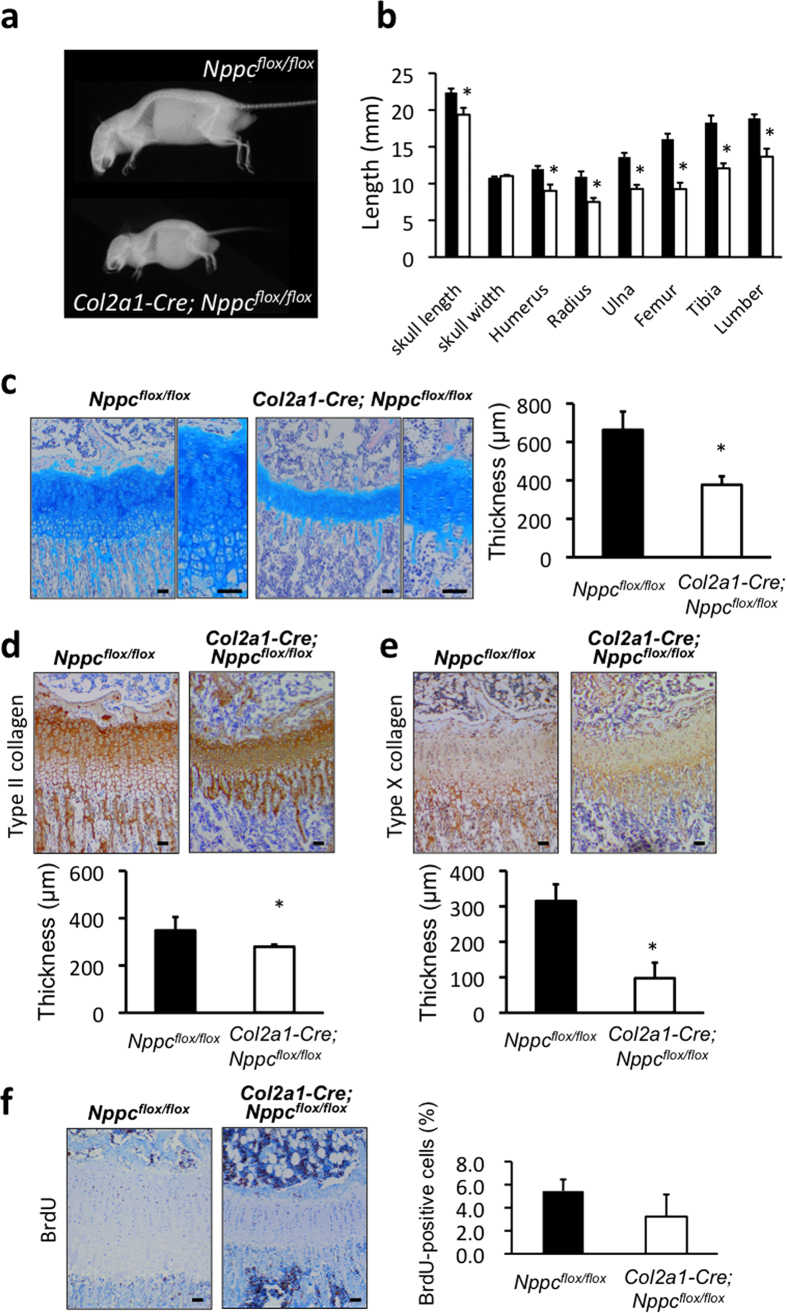
Impaired skeletal growth in *Col2a1-Cre; Nppc*^*flox/flox*^ mice. (**a**) Soft x-ray picture and (**b**) morphometric analyses of bones (skull length, skull width, humerus, radius, ulna, femur, tibia, and lumber) of *Nppc*^*flox/flox*^ and *Col2a1-Cre; Nppc*^*flox/flox*^ mice at the age of 10 weeks. *: P < 0.01, n = 5, each. (**c**)–(**f**) Histological analyses of tibial growth plates of *Nppc*^*flox/flox*^ and *Col2a1-Cre; Nppc*^*flox/flox*^ mice at the age of 2 weeks. Scale bar: 100 μm in each panel. (**c**) Micrographs of growth plates stained by Alcian Blue-hematoxylin and eosin (left pictures), and the thickness of growth plates (right graph). *: P < 0.01, n = 5, each. In micrographs, right panel in each set of panels is exhibited with higher magnification. (**d**), (**e**) Micrographs of tibial growth plates stained by type II (**d**) and X (**e**) collagen antibodies (upper pictures), and the thickness of nonhypertrophic (**d**) and hypertrophic (**e**) chondrocyte layers of the growth plates (lower graphs). *: P < 0.01, n = 5, each. (**f**) Micrographs of tibial growth plates with BrdU labeling (left pictures) and the proliferative rate of chondrocytes in growth plate shown as the average percent of BrdU-positive cells relative to total cell count (right graph).

Soft x-ray pictures revealed that the skeletal growth of *Col2a1–Cre; Nppc*^*flox/flox*^ mice was severely impaired (Fig. 2a). At 10 weeks, the lengths of all bones formed through endochondral ossification were significantly shorter in *Col2a1–Cre; Nppc*^*flox/flox*^ mice than in control mice: the lengths of humerus, radius, ulna, femur, tibia, and lumbar spine in *Col2a1–Cre; Nppc*^*flox/flox*^ mice were 75.2, 68.6, 68.1, 65.5, 65.9, and 72.3%, respectively, of the control lengths (Fig. 2b). The length of the skull, which is primarily defined by endochondral bone growth, was significantly shorter in *Col2a1–Cre; Nppc*^*flox/flox*^ mice at 86.5% of the control length. On the other hand, the width of the skull, which is defined by membranous ossification, did not differ between *Col2a1–Cre; Nppc*^*flox/flox*^ and control mice (Fig. 2b).

In order to further characterize the impaired growth of bones formed through endochondral ossification in cartilage-specific CNP knockout mice, we performed histological analyses of their growth plates. Histological images of the growth plate cartilage of proximal tibiae stained with Alcian Blue-hematoxylin and eosin revealed that at 2 weeks, the growth plates of *Col2a1–Cre; Nppc*^*flox/flox*^ mice were obviously and significantly thinner than those of control mice (Fig. 2c). As shown by immunohistochemical staining for type II collagen, which is a marker for the non-hypertrophic chondrocyte layer, the thickness of the non-hypertrophic chondrocyte layer in *Col2a1–Cre; Nppc*^*flox/flox*^ growth plates was 76.7% of the thickness in controls (Fig. 2d). Likewise, the extracellular spaces of the non-hypertrophic chondrocyte layer in *Col2a1–Cre; Nppc*^*flox/flox*^ growth plates were greatly decreased compared to those of control growth plates (Fig. 2d). Moreover, immunohistochemical staining for type X collagen, which is specifically expressed in the hypertrophic chondrocyte layer of the growth plate, revealed that the thickness of the hypertrophic chondrocyte layer in *Col2a1–Cre; Nppc*^*flox/flox*^ growth plates was drastically and significantly reduced, to 34.6% of the control thickness (Fig. 2e). The number and the size of hypertrophic chondrocytes in *Col2a1–Cre; Nppc*^*flox/flox*^ growth plates were dramatically decreased compared to those in the control growth plates.

**Figure 3:**
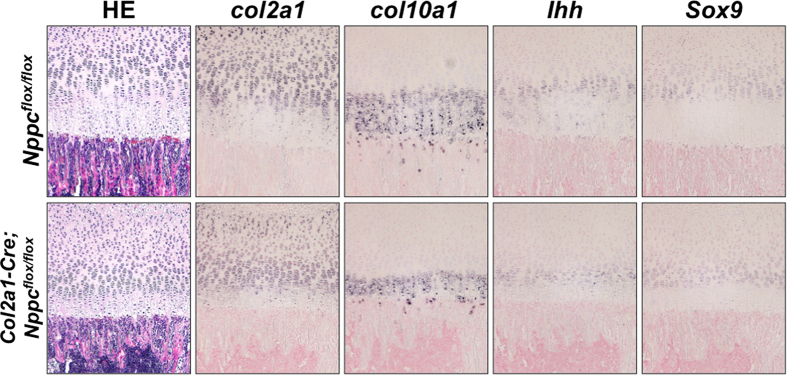
*In situ* hybridization analyses of the *Col2a1-Cre; Nppc*^*flox/flox*^ growth plate. *In situ* hybridization analyses for chondrocyte differentiation markers in the growth plates of *Nppc*^*flox/flox*^ and *Col2a1-Cre; Nppc*^*flox/flox*^ mice. HE; hematoxylin and eosin, Ihh; Indian hedgehog.

To investigate the decreased thickness of the proliferative chondrocyte layer of *Col2a1–Cre; Nppc*^*flox/flox*^ growth plates, we examined the proliferation of chondrocytes in this region by immunohistochemical staining for 5-bromo-2′-deoxy-uridine (BrdU) incorporation. The ratio of BrdU-positive chondrocytes tended to be lower in *Col2a1–Cre; Nppc*^*flox/flox*^ growth plates relative to control (Fig. 2f). To investigate the decreased thickness of the hypertrophic chondrocyte layer, we monitored apoptosis by terminal deoxynucleotidyl transferase-mediated dUTP nick-end labeling (TUNEL) staining. However, this assay revealed almost no difference in the extent of chondrocyte apoptosis between *Col2a1–Cre; Nppc*^*flox/flox*^ and control growth plates (Supplementary Fig. S3). Further we performed *in situ* hybridization analyses of differentiation markers of growth plate chondrocytes including type II and X collagens, Indian hedgehog and Sox9 (Fig. 3). We found the decreased areas expressing these differentiation markers in *Col2a1–Cre; Nppc*^*flox/flox*^ growth plates compared with those in control growth plates. Moreover, the intensities of these gene expressions, especially that of Indian hedgehog, tended to be lower in *Col2a1–Cre; Nppc*^*flox/flox*^ growth plates than in control growth plates.

### Effects of skeletal abnormalities on mortality of *Col2a1–Cre; Nppc* ^*flox/flox*^ mice

**Figure 4:**
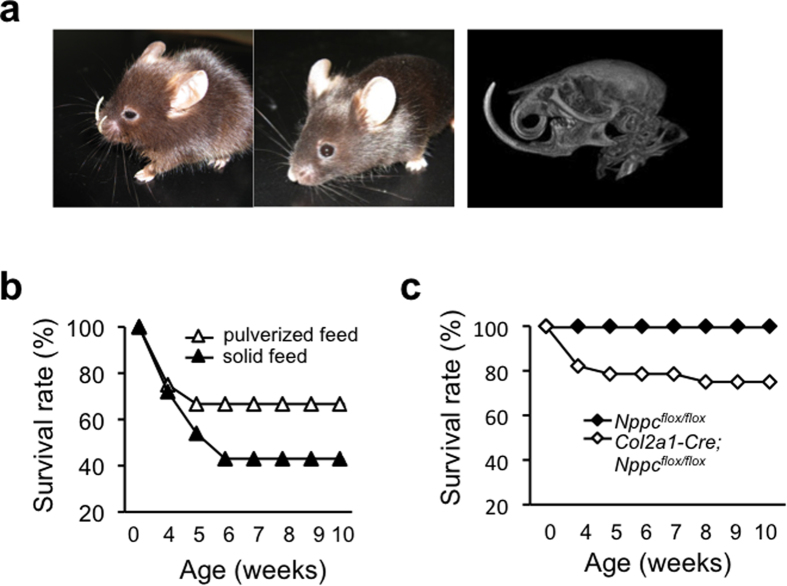
Effect of the skeletal abnormality in CNP mutant mice on mortality. (**a**) Left set of pictures: gross morphologies of 12-week-old total CNP knockout mice with severe malocclusion (left) and normal occlusion (right) of the incisal teeth. Right: three-dimensional reconstructed image of a 12-week-old *Nppc*^*−/−*^ mouse with severe malocclusion. (**b**) Survival curves of total CNP knockout mice supplied with solid feed (▲) and pulverized feed (Δ). (**c**) Survival curves of *Nppc*^*flox/flox*^ (Δ) and *Col2a1-Cre; Nppc*^*flox/flox*^ (◊) mice.

Previously, we reported that more than half of systemic CNP knockout mice die before adulthood^16^. A considerable proportion of total CNP knockout mice exhibit severe malocclusion, which may prevent normal eating (Fig. 4a); *Col2a1–Cre; Nppc*^*flox/flox*^ mice also exhibit this phenotype. In order to avoid starvation, we provided pulverized feed to total CNP knockout mice, and succeeded in increasing the survival rate (70% on pulverized feed vs. 40% on solid feed) (Fig. 4b). Subsequently, we provided pulverized feed to *Col2a1–Cre; Nppc*^*flox/flox*^ mice and examined the effect on survival rates. As shown by the Kaplan-Meier graphs depicted in Fig. 4c, the 10-week survival rates of *Col2a1–Cre; Nppc*^*flox/flox*^ mice were 75.0%.

### Generation and gross phenotypes of cartilage-specific GC-B knockout mice

**Figure 5:**
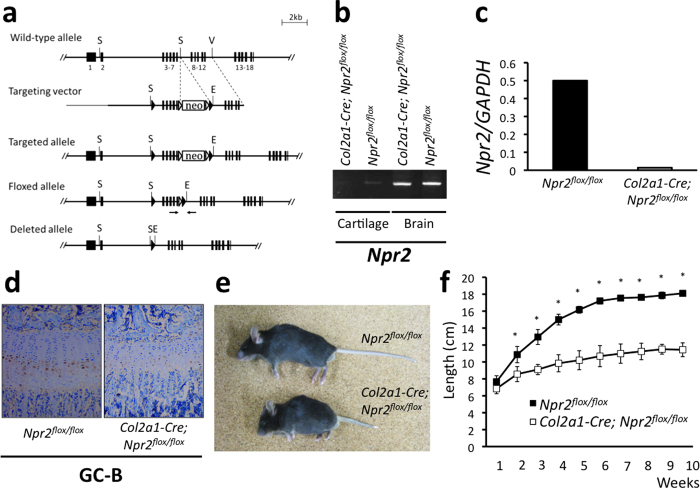
Generation and gross phenotype of *Col2a1-Cre; Npr2*^*flox/flox*^ mice. (**a**) Conditional targeting of *Npr2*. Restriction enzymes: E, *Eco*RI; S, *Sac*I; V, *Eco*RV; Arrows: PCR primers. Triangles (black): loxP sites. Triangles (white): FRT sites. (**b**) RT-PCR analysis of *Npr2* gene in cartilage and brain of *Col2a1-Cre; Npr2*^*flox/flox*^ and *Npr2*^*flox/flox*^ mice. The full-length gel is presented in Supplementary Figure S4. (**c**) Quantitative real-time RT-PCR analysis for GC-B expression in *Npr2*^*flox/flox*^ and *Col2a1-Cre; Npr2*^*flox/flox*^ mice. (**d**) Immunohistochemical analysis of GC-B in growth plates of *Npr2*^*flox/flox*^ and *Col2a1-Cre; Npr2*^*flox/flox*^ mice. (**e**) Gross appearance of *Npr2*^*flox/flox*^ (upper) and *Col2a1-Cre; Npr2*^*flox/flox*^ (lower) mice. (**f**) Growth curves of naso-tail length of *Npr2*^*flox/flox*^ (■) and *Col2a1-Cre; Npr2*^*flox/flox*^ (□) mice. *: P < 0.01.

We had previously reported that CNP and GC-B are expressed in nonhypertrophic and prehypertrophic chondrocyte layers of the growth plate, respectively^16^, i.e., GC-B exists in close proximity to CNP in the growth plate. This suggests a local regulation of endochondral bone growth in growth plate by the CNP/GC-B system. Therefore, likewise we performed targeted depletion of GC-B in murine cartilage using the Cre-loxP system, and further elucidated and confirmed the physiological effects of the local CNP/GC-B system in the growth plate on endochondral bone growth. Likewise, we generated mice carrying *Npr2*^*flox*^, an *Npr2* allele in which the DNA segment including exons 3–7 is flanked by *loxP* sites; exons 3–7 encode the C-terminal half of the extracellular ligand-binding domain and the transmembrane segment^17^ (Fig. 5a). Both heterozygous and homozygous mice carrying *Npr2*^*flox*^ were viable and fertile, and they exhibited no noticeable phenotypic change. Cartilage-specific *Npr2*-deleted mice (*Col2a1–Cre; Npr2*^*flox/flox*^) were generated by a breeding scheme similar to the one described above, in this case using heterozygous *Npr2*^*flox*^ and *Col2a1–Cre* transgenic mice. Cartilage-specific deletion of GC-B expression in *Col2a1–Cre; Nppc*^*flox/flox*^ mouse was confirmed by RT-PCR (Fig. 5b and Supplementary Fig. S4). Quantitative real-time RT-PCR analyses revealed that the expression of GC-B gene was almost completely depleted in the cartilage of *Col2a1–Cre; Npr2*^*flox/flox*^ mouse (Fig. 5c). The decrease of GC-B protein in the growth plate of *Col2a1–Cre; Npr2*^*flox/flox*^ mouse was exhibited by the immunohistochemical staining using anti-GC-B antibody (Fig. 5d).

We obtained qualitatively similar results from the cartilage-specific GC-B knockout (*Col2a1–Cre; Npr2*^*flox/flox*^) mice. At 10 weeks, *Col2a1–Cre; Npr2*^*flox/flox*^ mice were obviously shorter than *Npr2*^*flox/flox*^ mice (Fig. 5e). As shown by the growth curve, *Col2a1–Cre; Npr2*^*flox/flox*^ mice were slightly smaller than *Npr2*^*flox/flox*^ mice at 1 week; naso-anal and naso-tail lengths of *Col2a1–Cre; Npr2*^*flox/flox*^ mice were 90.7% and 89.3%, respectively, of the corresponding lengths in control mice (Fig. 5f and Supplementary Fig. S5). Later, the growth of *Col2a1–Cre; Npr2*^*flox/flox*^ mice was greatly retarded relative to controls: at 6 weeks, naso-anal and naso-tail lengths of *Col2a1–Cre; Npr2*^*flox/flox*^ mice were 73.8% and 62.1%, respectively, of the control lengths. After 6 weeks, *Col2a1–Cre; Npr2*^*flox/flox*^ mice grew similarly to the control mice, and at 10 weeks, the naso-anal and naso-tail lengths of *Col2a1–Cre; Npr2*^*flox/flox*^ mice were 72.5% and 63.1%, respectively, of the control lengths (Fig. 5f and Supplementary Fig. S5).

### Skeletal phenotypes of cartilage-specific GC-B knockout mice

**Figure 6:**
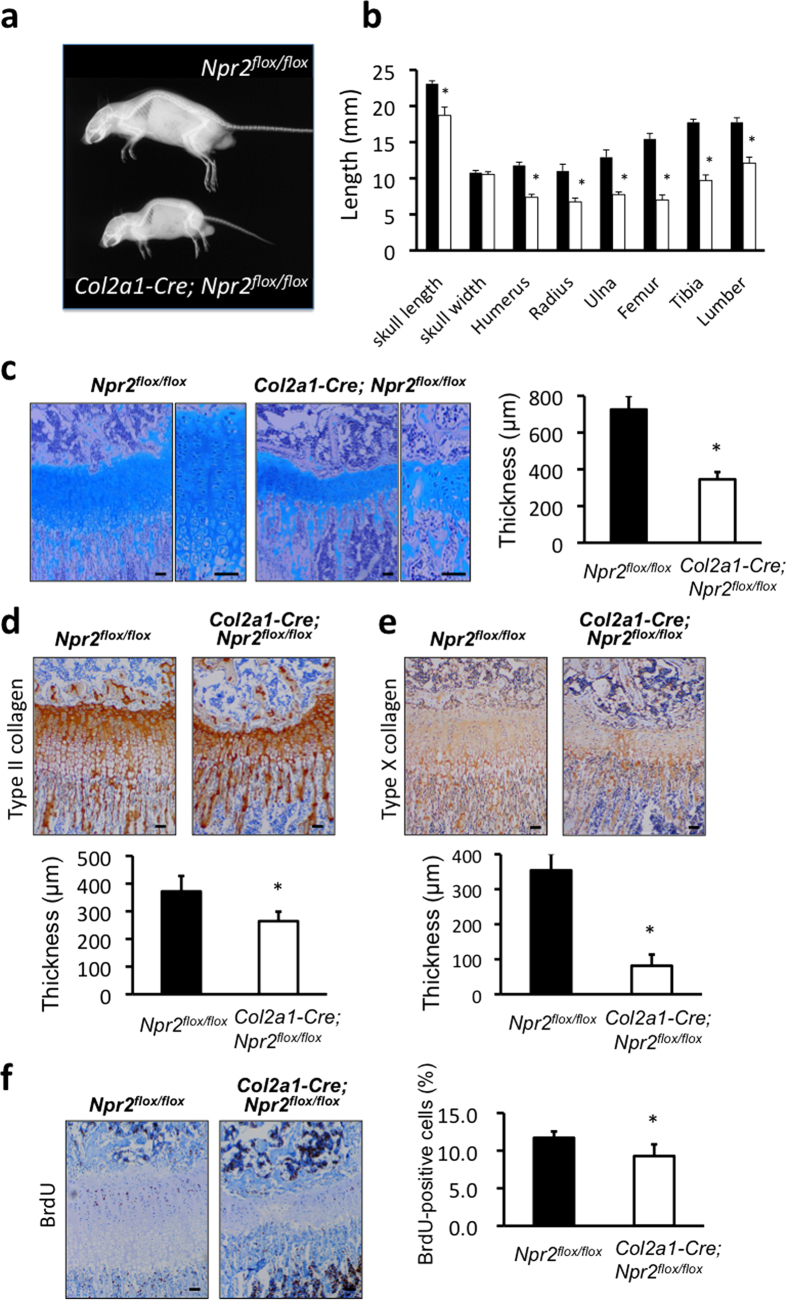
Impaired skeletal growth in *Col2a1-Cre; Npr2*^*flox/flox*^ mice. (**a**) Soft x-ray picture and (**b**) graph of lengths of bones (skull length, skull width, humerus, radius, ulna, femur, tibia, and lumber) of *Npr2*^*flox/flox*^ and *Col2a1-Cre; Npr2*^*flox/flox*^ mice at 10-week-old. *: P < 0.01, n = 5, each. (**c**)–(**f**) Histological analyses of tibial growth plates of *Npr2*^*flox/flox*^ and *Col2a1-Cre; Npr2*^*flox/flox*^ mice at the age of 2 weeks. Scale bar: 100 μm in each panel. (**c**) Micrographs of growth plates stained by Alcian Blue-hematoxylin and eosin (left pictures), and the thickness of growth plates (right graph). *: P < 0.01, n = 5, each. In micrographs, right panel in each set of panels is exhibited with higher magnification. (**d**), (**e**) Pictures of tibial growth plate stained by type II (**d**) and X (**e**) collagen antibodies (upper panels), and the thickness of nonhypertrophic (**d**) and hypertrophic (**e**) chondrocyte layers of the growth plates (lower graphs). *: P < 0.01, n = 5, each. (**f**) Pictures of tibial growth plates with BrdU staining (left panels) and the proliferative rate of chondrocytes in growth plate shown as the average percent of BrdU-positive cells relative to the total cell count (right graph). *: P < 0.05, n = 5, each.

As shown by soft x-ray pictures of mice at the age of 10 weeks, skeletal growth was severely impaired in *Col2a1–Cre; Npr2*^*flox/flox*^ mice (Fig. 6a). The lengths of humerus, radius, ulna, femur, tibia, and lumbar spine in *Col2a1–Cre; Npr2*^*flox/flox*^ mice were 62.4, 61.3, 60.0, 45.3, 54.6, and 68.2%, respectively, of their lengths in control mice (Fig. 6b). The length of the skull was significantly shorter in *Col2a1–Cre; Npr2*^*flox/flox*^, at 81.0% of the control length, but the width of the skull did not differ between *Col2a1–Cre; Npr2*^*flox/flox*^ and control mice (Fig. 6b). In addition, *Col2a1–Cre; Npr2*^*flox/flox*^ mice also exhibited malocclusion just as observed in systemic or cartilage-specific CNP knockout mice.

Further we performed histological analyses of the growth plates of cartilage-specific GC-B knockout mice. As in the case of *Col2a1–Cre; Nppc*^*flox/flox*^ mice, the growth-plate cartilage of the proximal tibiae in *Col2a1–Cre; Npr2*^*flox/flox*^ mice was obviously and significantly thinner than in control mice (Fig. 6c). Immunohistochemical staining for type II collagen revealed that the thickness of the non-hypertrophic chondrocyte layer in *Col2a1–Cre; Npr2*^*flox/flox*^ growth plates was 71.1% of the thickness in controls (Fig. 6d). The extracellular spaces of the non-hypertrophic chondrocyte layer in *Col2a1–Cre; Npr2*^*flox/flox*^ growth plates were greatly decreased compared to those of controls (Fig. 6d). Immunohistochemical staining for type X collagen revealed that the thickness of the hypertrophic chondrocyte layer in *Col2a1–Cre; Npr2*^*flox/flox*^ growth plates was severely decreased, to 23.0% of the control thickness (Fig. 6e). The number and the size of hypertrophic chondrocytes in *Col2a1–Cre; Npr2*^*flox/flox*^ growth plates were greatly decreased compared to those in the control growth plates. Immunohistochemical staining for BrdU incorporation of the proliferative chondrocyte layer of *Col2a1–Cre; Npr2*^*flox/flox*^ growth plates revealed that the ratio of BrdU-positive chondrocytes was significantly lower in *Col2a1–Cre; Npr2*^*flox/flox*^ growth plates relative to control (Fig. 6f). As in the case of *Col2a1–Cre; Nppc*^*flox/flox*^ mice, TUNEL staining revealed almost no difference in the extent of chondrocyte apoptosis between *Col2a1–Cre; Npr2*^*flox/flox*^ and control growth plates (Supplementary Fig. S6).

## Discussion

The CNP/GC-B system is present in a wide variety of tissues and has been assumed to play roles as a local regulator^22^. CNP was first isolated from porcine brain^23^ and is distributed throughout the brain and pituitary^6^,^8^,^24^,^25^. In the cardiovascular system, CNP is expressed in endothelial cells, where it acts as a vascular mediator regulating local vascular tone and growth^9^,^10^,^26^,^27^. In ovarian maturation, the CNP/GC-B system is essential for oocyte meiotic arrest and cumulus oophorus formation^11^,^12^,^13^,^28^,^29^,^30^. As for skeletal tissue, we had elucidated that the CNP/GC-B system directly stimulates endochondral bone growth in the growth plate; CNP stimulates tibial explants from fetal mice in organ culture^31^, and mice with targeted overexpression of CNP in the growth plate exhibit prominent skeletal overgrowth phenotype^32^. Indeed, CNP and GC-B exist in nonhypertrophic and prehypertrophic chondrocyte layers of the growth plate, respectively^16^. Together with the fact that mice with systemic depletion of CNP or GC-B exhibit severely impaired growth of bones formed through endochondral ossification, we could suppose that the local CNP/GC-B system in the growth plate is a physiological stimulator of endochondral bone growth. Nevertheless, it remains possible that the CNP/GC-B physiologically regulates endochondral bone growth via mechanisms other than the local effect on the growth plate; CNP secreted from a tissue other than growth plate cartilage might influence or stimulate endochondral bone growth. In fact, CNP is capable of humorally stimulating endochondral bone growth, as demonstrated by the observation that transgenic mice with elevated plasma concentrations of CNP exhibit skeletal overgrowth phenotype^20^,^33^. Furthermore, in tissues other than growth-plate cartilage, the CNP/GC-B system may stimulate other regulatory systems that influence endochondral bone growth, e.g., the GH/IGF-I axis. In this study, however, we showed that the extent of impairment of endochondral bone growth observed in cartilage-specific CNP or GC-B knockout mice is almost the same as in systemic CNP or GC-B knockout mice, respectively. Thus, the autocrine/paracrine effect of the CNP/GC-B system in the growth plate is the primary physiological stimulator of endochondral bone growth in body.

In the present study, first we generated mice with cartilage-specific depletion of CNP, the ligand of the CNP/GC-B system (*Col2a1-Cre; Nppc*^*flox/flox*^ mice). Next, to further confirm the local regulation of endochondral bone growth by the system, we developed mice with cartilage-specific depletion of the receptor, GC-B (*Col2a1-Cre; Npr2*^*flox/flox*^ mice). As the result, we could obtain qualitatively the same skeletal phenotype in *Col2a1-Cre; Nppc*^*flox/flox*^ and *Col2a1-Cre; Npr2*^*flox/flox*^ mice, strengthening the notion that the CNP/GC-B system is the local stimulator of physiological endochondral bone growth. Nevertheless, impairment of skeletal growth in *Col2a1-Cre; Npr2*^*flox/flox*^ mice was more severe than in *Col2a1-Cre; Nppc*^*flox/flox*^ mice. The most plausible reason for this discrepancy would be that in *Col2a1-Cre; Nppc*^*flox/flox*^ mice, CNP secreted from tissues other than cartilage (e.g., blood vessels), could bind GC-B in the growth plates, resulting in a milder impairment of endochondral bone growth than in *Col2a1-Cre; Npr2*^*flox/flox*^ mice. Furthermore, as the abundance of a ligand is greater than that of its receptor in general, the amount of the leaked ligand, CNP, in *Col2a1-Cre; Nppc*^*flox/flox*^ mice might be greater than that of its receptor (GC-B) in *Col2a1-Cre; Npr2*^*flox/flox*^ mice, so the leaking effect of the CNP/GC-B system on endochondral could be stronger in *Col2a1-Cre; Nppc*^*flox/flox*^ mice than in *Col2a1-Cre; Npr2*^*flox/flox*^ mice. In addition, this might be because in the absence of CNP, the sensitivity of GC-B may be increased or a clearance system of natriuretic peptide may be inactivated (e.g., through the down-regulation of the expression of C-receptor^33^, or by increasing the natural ligand of C-receptor, osteocrin^34^), and therefore GC-B may easily be activated by other natriuretic peptides, i.e., ANP or BNP. Actually, increased circulating BNP can crossreact with GC-B and stimulate endochondral bone growth in mice^35^,^36^.

Histological images revealed that among the chondrocyte layers of the growth plate, the hypertrophic chondrocyte layers were the most drastically reduced in both *Col2a1-Cre; Nppc*^*flox/flox*^ and *Col2a1-Cre; Npr2*^*flox/flox*^ mice. *In situ* hybridization analyses of *Col2a1-Cre; Nppc*^*flox/flox*^ growth plate revealed the decreased areas of the gene expressions for chondrocyte differentiation markers and tendencies of decreased intensities of these gene expressions, especially that of Indian hedgehog, a key molecule for chondrocyte differentiation that plays an important role on the transition from prehypertrophic chondrocytes to hypertrophic chondrocytes. In addition, the height of the proliferative chondrocyte layer, along with the proliferation of chondrocytes in that region, was moderately reduced in both *Col2a1-Cre; Nppc*^*flox/flox*^ and *Col2a1-Cre; Npr2*^*flox/flox*^ growth plates. These data indicate that under physiological conditions, the CNP/GC-B system in the growth plate mildly stimulates the proliferation of chondrocytes in the proliferative chondrocyte layer, and then potently promotes their hypertrophic differentiation; the promotion of the transitional differentiation from prehypertrophic chondrocytes to hypertrophic chondrocytes is thought to be the major contributing factor for the stimulatory effect of CNP/GC-B signaling on endochondral bone growth. As for the mechanism of the strong and physiological stimulatory effect of hypertrophic differentiation by the CNP/GC-B system, Kawasaki *et al.* exhibited that a downstream molecule of the CNP/GC-B system, cGMP dependent protein kinase (cGK) II^37^, promotes chondrocyte hypertrophy through phosphorylation of glycogen synthase kinase-3β and following decreased degradation of β-catenin^38^. This explanation sounds plausible, however, there still remains elusive problem that the narrowed growth plate of CNP or GC-B knockout is quite different from the extraordinary widened growth plate of cGKII knockout mice. Further studies must be necessary for the clarification of this point.

Previously, we reported that the survival rate of total CNP knockout mice is about 40%^16^. We hypothesized that one reason for the early death of these mice after weaning is starvation caused by their severe malocclusion. Indeed, the survival rate of total CNP knockout mice increased to ~70% when they were supplied with pulverized feed. Likewise, the survival rate of *Col2a1-Cre; Nppc*^*flox/flox*^ mice on pulverized feed (75.0%) is almost the same as that of total CNP knockout mice on pulverized feed. These data indicate that the increased prevalence of early death in total CNP knockout mice is caused by their impaired skeletal growth. However, the mechanisms by which the impaired skeletal growth of these knockout mice increases their mortality (to ~30%) are still unknown. Further experiments to address this issue are now ongoing in our laboratory.

In conclusion, we have revealed that the CNP/GC-B system is responsible for physiological endochondral bone growth via local action in cartilage, even though the CNP/GC-B system is ubiquitously present. Furthermore, this pathway might further affect mortality via unknown mechanisms.

## Material and Methods

### Ethics statement

Animal care and all experiments were conducted in accordance with the institutional guidelines of Kyoto University Graduate School of Medicine. All experimental protocols were approved by the institutional committee of Kyoto University Graduate School of Medicine.

### Generation of *Nppc* ^*flox*^ mice and *Npr2* ^*flox*^ mice

The targeting vector contains two flippase recombination target (FRT) sites that flank neomycin cassettes, and two *loxP* sites that flank exons 1 and 2 of *Nppc* for *Nppc*^*flox*^ mice or exons 3–7 of *Npr2* for *Npr2*^*flox*^ mice and neomycin cassettes (Fig.1a, 4a). Linearized targeting vector was transfected into embryonic stem (ES) cells. ES clones heterozygous for the *Nppc-*floxed or *Npr2*-floxed allele were identified by PCR screening and confirmed by Southern-blot analysis using an *Nppc* or *Npr2* probe, respectively, located outside of the homology regions used for gene recombination. Mouse chimeras were generated by C57BL/6 host blastocyst injection of mutant ES-cell clones, and the resulting chimeras were bred with C57BL/6 mice to generate *Nppc*-floxed or *Npr2*-floxed heterozygous mice.

### Generation of cartilage-specific *Nppc* knockout mice and cartilage-specific *Npr2* knockout mice

In the first cross, *Col2a1–Cre* transgenic mice^21^,^39^ were mated with mice heterozygous for the *Nppc*-floxed or *Npr2*-floxed allele. Offspring inheriting the *Col2a1–Cre* and *Nppc*-floxed or *Npr2*-floxed alleles were then mated with *Nppc*-floxed or *Npr2*-floxed heterozygous mice to obtain embryos harboring the *Col2a1–Cre* transgene along with two *Nppc*-floxed or *Npr2*-floxed alleles. Routine mouse genotyping was performed by PCR. The following primer pairs were used: *Cre*, 5′-TCCAATTTACTGACCGTACACCAA-3′ and 5′-CCTGATCCTGGCAATTTCGGCTA3′; *Nppc* floxdel allele, 5′-GTGTCCACAGTGAGTTCTTTACCAG-3′ and 5′-GTAAAGTGTGTCTCATCATCACATCATC-3′; and *Npr2* floxdel allele, 5′-GTAACCTGGGTAGACTAGTTGTTGG-3′ and 5′-ATGGTGGAGGAGGTCTTTAATTCC-3.

There was not any gender difference in the results of this study, so we presented the results of experiments using male mice only as the representative.

### Quantification of the gene expressions for *Nppc* and *Npr2*

For quantification of the gene expressions for *Nppc* and *Npr2*, real-time RT-PCR was performed in a StepOne™ real-time PCR System (Applied Biosystems). Complementary DNA was mixed with TaqMan® Universal PCR Master Mix (Applied Biosystems) and TaqMan® Gene Expression Assay primers (Applied Biosystems): natriuretic peptide receptor 2 (Mm00612889_m1), natriuretic peptide type C (Mm01295410_m1), and GAPDH (Mm99999915_g1). All RNA samples were titrated to yield equal amplification of GAPDH as an internal normalization control. Reactions for each sample were performed in triplicate. After an initial denaturation step (95 °C for 10 min), amplification was performed for 45 cycles (15-second denaturation at 95 °C and 60-second extension at 60 °C).

### Skeletal analysis

Skeletal analysis was performed as previously described^32^. Briefly, mice were subjected to soft x-ray analysis (30 kVp, 5 mA for 1 min; Softron Type SRO-M5; Softron, Tokyo, Japan), and the lengths of the bones were measured on the soft x-ray film. Naso-tail and naso-anal length measurements were made every week.

### Histological analysis

For light microscopy, sections were cut from paraffin-embedded specimens. For Alcian Blue-hematoxylin and eosin staining, sections were deparaffinized with xylene and rehydrated through an ethanol series and distilled water. The sections were treated with 3% acetic acid for 3 min, and with Alcian Blue (Muto Pure Chemicals Co., Ltd., Tokyo, Japan) for 20 min. Next, they were treated with hematoxylin (Muto) for 2 min, eosin alcohol (Muto) for 1 min, dehydrated, and then mounted with malinol (Muto).

For immunohistochemical detection of CNP, GC-B, type II collagen, and type X collagen, tissue sections were incubated with monoclonal anti-CNP antibody (T-4223, Peninsula Laboratories, CA, USA), anti-GC-B antibody (sc-16870, Santa Cruz Biotechnology, Santa Cruz, CA, USA), type II collagen antibody (1320-01, Southern Biotech, AL, USA), or type X collagen antibody (LB-0092, LSL, Japan), and immunostaining was performed using the Histofine MOUSESTAIN kit (Nichirei Corp., Tokyo, Japan) according to the manufacturer’s instructions.

*In situ* hybridization analyses were performed as previously described in Reference 20.

### BrdU analysis of growth plates

BrdU (5-Bromo-2′-deoxyuridine, 05650, Nacalai, Kyoto, Japan) was injected intraperitoneally at a concentration of 50 |g/g body weight 2 h before sacrifice. Target skeletal tissues were harvested, fixed overnight at 4 °C in a 4% paraformaldehyde solution, and then decalcified for 2 weeks in 0.5 M EDTA. Decalcified samples were embedded in paraffin and sectioned. BrdU-positive cells were detected using a BrdU antibody. The number of BrdU-positive nuclei, as a percentage of the total number of nuclei, was defined as the proliferation index.

### Genotype-specific survival

Generation of total CNP knockout mice (*Nppc*^*−/−*^ mice) was previously reported^16^. After weaning, *Nppc*^−/−^ mice were divided into one group that was provided with solid feed and another group that was provided with pulverized feed. *Col2a1–Cre; Nppc*^*flox/flox*^ mice were all provided with pulverized feed. Differences in survival rates between genotypes were assessed using Kaplan-Meier analysis.

### Statistical analysis

Data are expressed as means ± SEM. Statistical analysis was performed using ANOVA with Fisher’s least-significant-difference method when appropriate. P values less than 0.05 were considered statistically significant.

## Additional Information

**How to cite this article**: Nakao, K. *et al*. The Local CNP/GC-B system in growth plate is responsible for physiological endochondral bone growth. *Sci. Rep.* **5**, 10554; doi: 10.1038/srep10554 (2015).

## Supplementary Material

Supplementary Information

## References

[b1] Nakao K., Ogawa Y., Suga S. & Imura H. Molecular biology and biochemistry of the natriuretic peptide system. I: Natriuretic peptides. J Hypertens 10, 907–912 (1992). 1328371

[b2] Nakao K., Ogawa Y., Suga S. & Imura H. Molecular biology and biochemistry of the natriuretic peptide system. II: Natriuretic peptide receptors. J Hypertens 10, 1111–1114 (1992). 1334991 10.1097/00004872-199210000-00002

[b3] Suga S. *et al.* Receptor selectivity of natriuretic peptide family, atrial natriuretic peptide, brain natriuretic peptide, and C-type natriuretic peptide. Endocrinology 130, 229–239 (1992). 1309330 10.1210/endo.130.1.1309330

[b4] Pandey K. N., Oliver P. M., Maeda N. & Smithies O. Hypertension associated with decreased testosterone levels in natriuretic peptide receptor-A gene-knockout and gene-duplicated mutant mouse models. Endocrinology 140, 5112–5119 (1999). 10.1210/endo.140.11.7121 10537139

[b5] Ueda S. *et al.* Distribution and characterization of immunoreactive porcine C-type natriuretic peptide. Biochem Biophys Res Commun 175, 759–767 (1991). 1827257 10.1016/0006-291x(91)91631-l

[b6] Komatsu Y. *et al.* C-type natriuretic peptide (CNP) in rats and humans. Endocrinology 129, 1104–1106 (1991). 1855454 10.1210/endo-129-2-1104

[b7] Yandle T. G., Fisher S., Charles C., Espiner E. A. & Richards A. M. The ovine hypothalamus and pituitary have markedly different distribution of C-type natriuretic peptide forms. Peptides 14, 713–716 (1993). 8234014 10.1016/0196-9781(93)90102-m

[b8] Minamino N. *et al.* Distribution of C-type natriuretic peptide and its messenger RNA in rat central nervous system and peripheral tissue. Biochem Biophys Res Commun 197, 326–335 (1993). 8250942 10.1006/bbrc.1993.2479

[b9] Suga S. *et al.* Cytokine-induced C-type natriuretic peptide (CNP) secretion from vascular endothelial cells--evidence for CNP as a novel autocrine/paracrine regulator from endothelial cells. Endocrinology 133, 3038–3041 (1993). 8243333 10.1210/endo.133.6.8243333

[b10] Komatsu Y. *et al.* Regulation of endothelial production of C-type natriuretic peptide in coculture with vascular smooth muscle cells. Role of the vascular natriuretic peptide system in vascular growth inhibition. Circ Res 78, 606–614 (1996). 8635218 10.1161/01.res.78.4.606

[b11] Zhang M., Su Y. Q., Sugiura K., Xia G. & Eppig J. J. Granulosa cell ligand NPPC and its receptor NPR2 maintain meiotic arrest in mouse oocytes. Science 330, 366–369 (2010). 20947764 10.1126/science.1193573 PMC3056542

[b12] Zhang M. *et al.* Estradiol promotes and maintains cumulus cell expression of natriuretic peptide receptor 2 (NPR2) and meiotic arrest in mouse oocytes *in vitro*. Endocrinology 152, 4377–4385 (2011). 21914782 10.1210/en.2011-1118 PMC3199003

[b13] Kiyosu C., Tsuji T., Yamada K., Kajita S. & Kunieda T. NPPC/NPR2 signaling is essential for oocyte meiotic arrest and cumulus oophorus formation during follicular development in the mouse ovary. Reproduction 144, 187–193 (2012). 22696190 10.1530/REP-12-0050

[b14] Middendorff R., Muller D., Paust H. J., Davidoff M. S. & Mukhopadhyay A. K. Natriuretic peptides in the human testis: evidence for a potential role of C-type natriuretic peptide in Leydig cells. J Clin Endocrinol Metab 81, 4324–4328 (1996). 8954035 10.1210/jcem.81.12.8954035

[b15] Xia W., Mruk D. D. & Cheng C. Y. C-type natriuretic peptide regulates blood-testis barrier dynamics in adult rat testes. Proc Natl Acad Sci U S A 104, 3841–3846 (2007). 17360440 10.1073/pnas.0610100104 PMC1820671

[b16] Chusho H. *et al.* Dwarfism and early death in mice lacking C-type natriuretic peptide. Proc Natl Acad Sci U S A 98, 4016–4021 (2001). 11259675 10.1073/pnas.071389098 PMC31171

[b17] Tamura N. *et al.* Critical roles of the guanylyl cyclase B receptor in endochondral ossification and development of female reproductive organs. Proc Natl Acad Sci U S A 101, 17300–17305 (2004). 15572448 10.1073/pnas.0407894101 PMC534612

[b18] Nakao K. *et al.* The effects of C-type natriuretic peptide on craniofacial skeletogenesis. J Dent Res 92, 58–64 (2013). 23114031 10.1177/0022034512466413

[b19] Lupu F., Terwilliger J. D., Lee K., Segre G. V. & Efstratiadis A. Roles of growth hormone and insulin-like growth factor 1 in mouse postnatal growth. Dev Biol 229, 141–162 (2001). 11133160 10.1006/dbio.2000.9975

[b20] Fujii T. *et al.* Circulating C-type natriuretic peptide (CNP) rescues chondrodysplastic CNP knockout mice from their impaired skeletal growth and early death. Endocrinology 151, 4381–4388 (2010). 20610569 10.1210/en.2010-0078

[b21] Ovchinnikov D. A., Deng J. M., Ogunrinu G. & Behringer R. R. Col2a1-directed expression of Cre recombinase in differentiating chondrocytes in transgenic mice. Genesis 26, 145–146 (2000). 10686612

[b22] Potter L. R. Guanylyl cyclase structure, function and regulation. Cell Signal 23, 1921–1926 (2011). 21914472 10.1016/j.cellsig.2011.09.001 PMC4856045

[b23] Sudoh T., Minamino N., Kangawa K. & Matsuo H. C-type natriuretic peptide (CNP): a new member of natriuretic peptide family identified in porcine brain. Biochem Biophys Res Commun 168, 863–870 (1990). 2139780 10.1016/0006-291x(90)92401-k

[b24] Porzionato A., Macchi V., Rucinski M., Malendowicz L. K. & De Caro R. Natriuretic peptides in the regulation of the hypothalamic-pituitary-adrenal axis. Int Rev Cell Mol Biol 280, 1–39 (2010). 20797680 10.1016/S1937-6448(10)80001-2

[b25] Thompson I. R. *et al.* Expression of guanylyl cyclase-B (GC-B/NPR2) receptors in normal human fetal pituitaries and human pituitary adenomas implicates a role for C-type natriuretic peptide. Endocr Relat Cancer 19, 497–508 (2012). 22645228 10.1530/ERC-12-0129

[b26] Suga S. *et al.* Endothelial production of C-type natriuretic peptide and its marked augmentation by transforming growth factor-beta. Possible existence of “vascular natriuretic peptide system”. J Clin Invest 90, 1145–1149 (1992). 1522222 10.1172/JCI115933 PMC329977

[b27] Suga S. I. *et al.* Regulation of endothelial production of C-type natriuretic peptide by interaction between endothelial cells and macrophages. Endocrinology 139, 1920–1926 (1998). 9528978 10.1210/endo.139.4.5918

[b28] Kawamura K. *et al.* Pre-ovulatory LH/hCG surge decreases C-type natriuretic peptide secretion by ovarian granulosa cells to promote meiotic resumption of pre-ovulatory oocytes. Hum Reprod 26, 3094–3101 (2011). 21865234 10.1093/humrep/der282

[b29] Robinson J. W. *et al.* Luteinizing hormone reduces the activity of the NPR2 guanylyl cyclase in mouse ovarian follicles, contributing to the cyclic GMP decrease that promotes resumption of meiosis in oocytes. Dev Biol 366, 308–316 (2012). 22546688 10.1016/j.ydbio.2012.04.019 PMC3358460

[b30] Sato Y., Cheng Y., Kawamura K., Takae S. & Hsueh A. J. C-type natriuretic peptide stimulates ovarian follicle development. Mol Endocrinol 26, 1158–1166 (2012). 22595960 10.1210/me.2012-1027 PMC3385790

[b31] Yasoda A. *et al.* Natriuretic peptide regulation of endochondral ossification. Evidence for possible roles of the C-type natriuretic peptide/guanylyl cyclase-B pathway. J Biol Chem 273, 11695–11700 (1998). 9565590 10.1074/jbc.273.19.11695

[b32] Yasoda A. *et al.* Overexpression of CNP in chondrocytes rescues achondroplasia through a MAPK-dependent pathway. Nat Med 10, 80–86 (2004). 14702637 10.1038/nm971

[b33] Kake T. *et al.* Chronically elevated plasma C-type natriuretic peptide level stimulates skeletal growth in transgenic mice. Am J Physiol Endocrinol Metab 297, E1339–1348 (2009). 19808910 10.1152/ajpendo.00272.2009

[b34] Moffatt P. *et al.* Osteocrin is a specific ligand of the natriuretic Peptide clearance receptor that modulates bone growth. J Biol Chem 282, 36454–36462 (2007). 17951249 10.1074/jbc.M708596200

[b35] Suda M. *et al.* Skeletal overgrowth in transgenic mice that overexpress brain natriuretic peptide. Proc Natl Acad Sci U S A 95, 2337–2342 (1998). 9482886 10.1073/pnas.95.5.2337 PMC19337

[b36] Chusho H. *et al.* Genetic models reveal that brain natriuretic peptide can signal through different tissue-specific receptor-mediated pathways. Endocrinology 141, 3807–3813 (2000). 11014237 10.1210/endo.141.10.7692

[b37] Miyazawa T. *et al.* Cyclic GMP-dependent protein kinase II plays a critical role in C-type natriuretic peptide-mediated endochondral ossification. Endocrinology 143, 3604–3610 (2002). 12193576 10.1210/en.2002-220307

[b38] Kawasaki Y. *et al.* Phosphorylation of GSK-3beta by cGMP-dependent protein kinase II promotes hypertrophic differentiation of murine chondrocytes. J Clin Invest 118, 2506–2515 (2008). 18551195 10.1172/JCI35243 PMC2423867

[b39] Akiyama H. *et al.* Interactions between Sox9 and beta-catenin control chondrocyte differentiation. Genes Dev 18, 1072–1087 (2004). 15132997 10.1101/gad.1171104 PMC406296

