# Supplementary material for: The Local CNP/GC-B system in growth plate is responsible for physiological endochondral bone growth
Source: Sci Rep. 2015 May 27;5:10554. doi: 10.1038/srep10554 (PMC5395013; doi:10.1038/srep10554)
Supplement: Supplementary Information [file srep10554-s1.pdf]

Supplementary Information

# The Local CNP/GC-B system in growth plate is responsible for physiological endochondral bone growth

Kazumasa Nakao[1], Kenji Osawa[1], Akihiro Yasoda[2], Shigeki Yamanaka[1], Toshihito Fujii[2], Eri Kondo[2], Noriaki Koyama[1], Naotetsu Kanamoto[2], Masako Miura[2], Koichiro Kuwahara[3], Haruhiko Akiyama[4], Kazuhisa Bessho[1] and Kazuwa Nakao[5]

[1]Department of Oral and Maxillofacial Surgery, Kyoto University Graduate School of Medicine, Kyoto 606-8507, Japan
[2]Department of Diabetes, Endocrinology and Nutrition, Kyoto University Graduate School of Medicine, Kyoto 606-8507, Japan
[3]Department of Cardiovascular Medicine, Kyoto University Graduate School of Medicine, Kyoto 606-8507, Japan
[4]Department of Orthopaedic Surgery, Gifu University Graduate School of Medicine, Gifu 501-1194, Japan
[5]Medical Innovation Center, Kyoto University Graduate School of Medicine, Kyoto 606-8507, Japan

Supplementary Figure

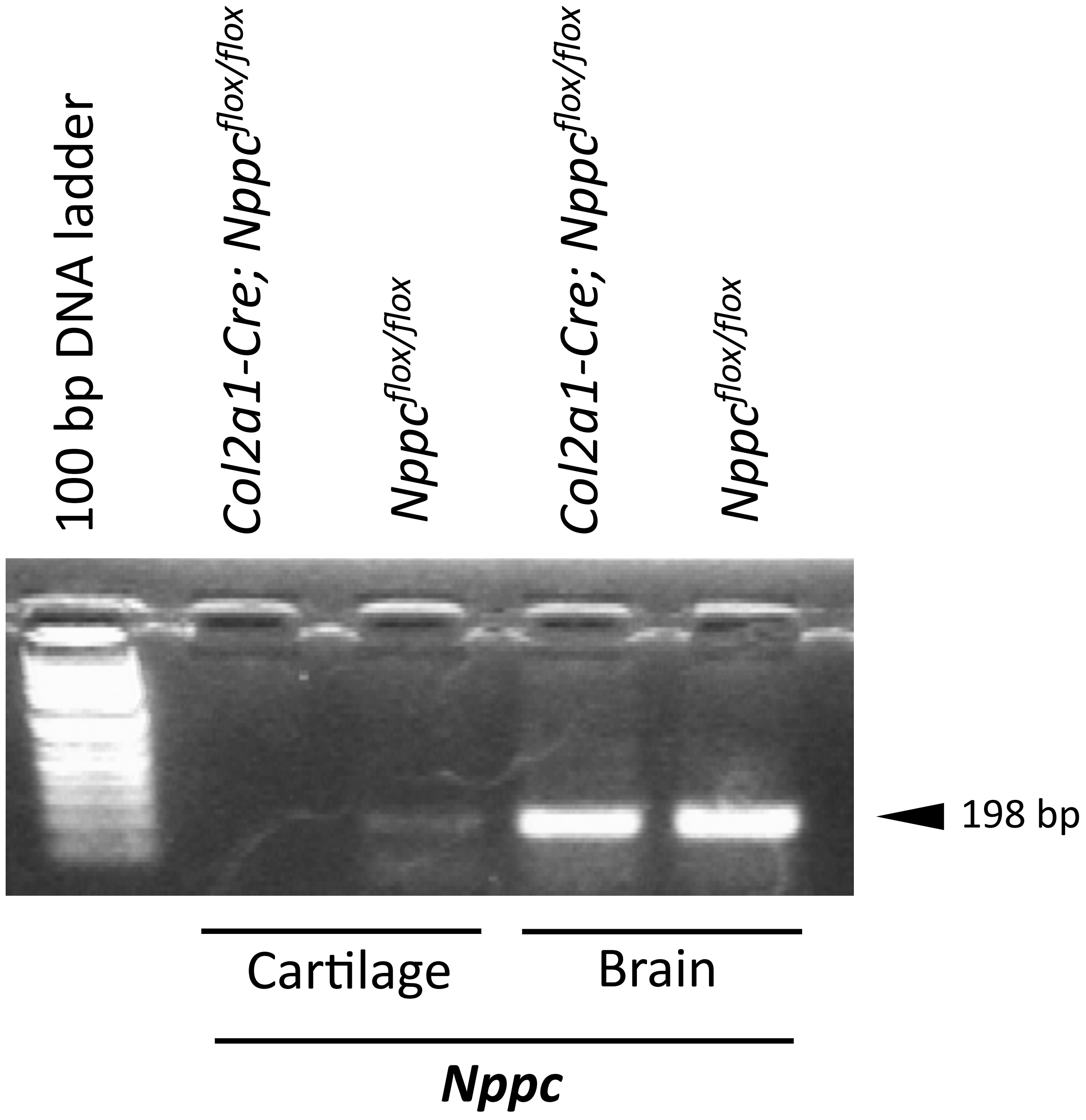


**Supplementary Figure S1**

**The full-length gel exhibiting the result of the RT-PCR for *Nppc* in cartilage and brain of *Col2-Cre;* $Nppc^{flox/flox}$ and $Nppc^{flox/flox}$ mice.**

Supplementary Figure

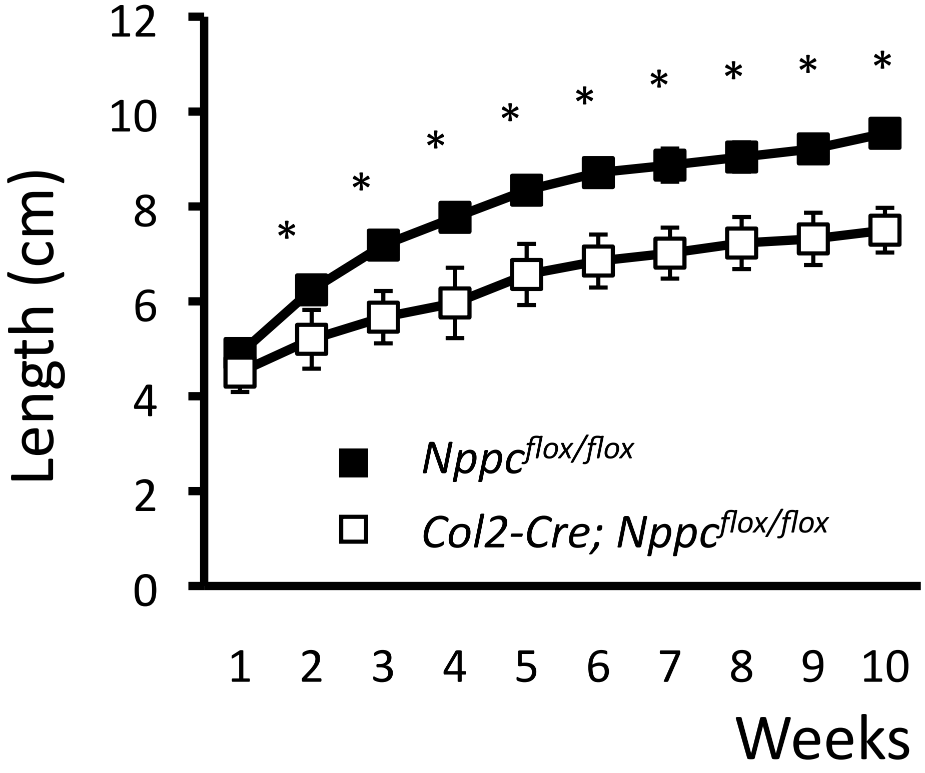


**Supplementary Figure S2**

**Growth curves of naso-anal length of *Nppc*$^{\textit{flox/flox}}$ (■) and *Col2-Cre; Nppc*$^{\textit{flox/flox}}$ (□) mice.**

*: P < 0.01.

Supplementary Figure

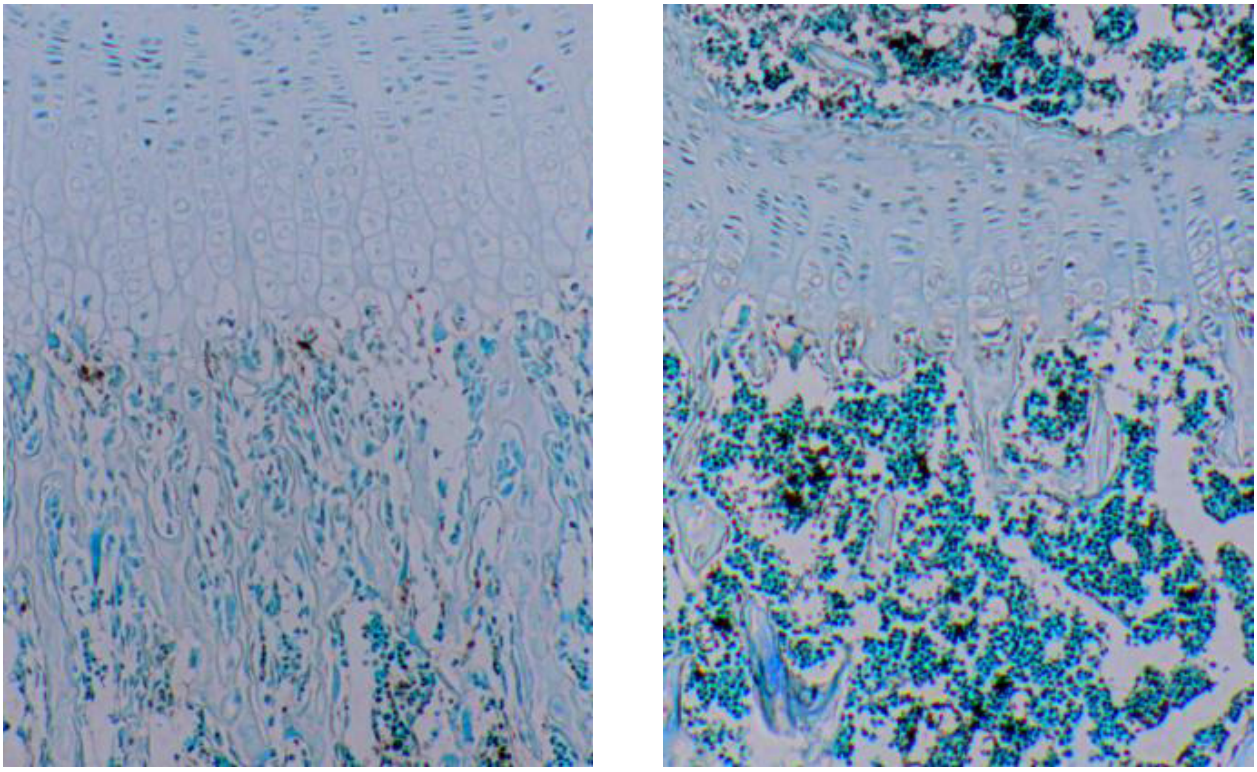

*Nppc*$^{flox/flox}$ *Col2a1-Cre; Nppc*$^{flox/flox}$

**Supplementary Figure S3**
**TUNEL staining of growth plates of *Nppc*$^{flox/flox}$ and *Col2-Cre; Npr2*$^{flox/flox}$ mice.**

Supplementary Figure

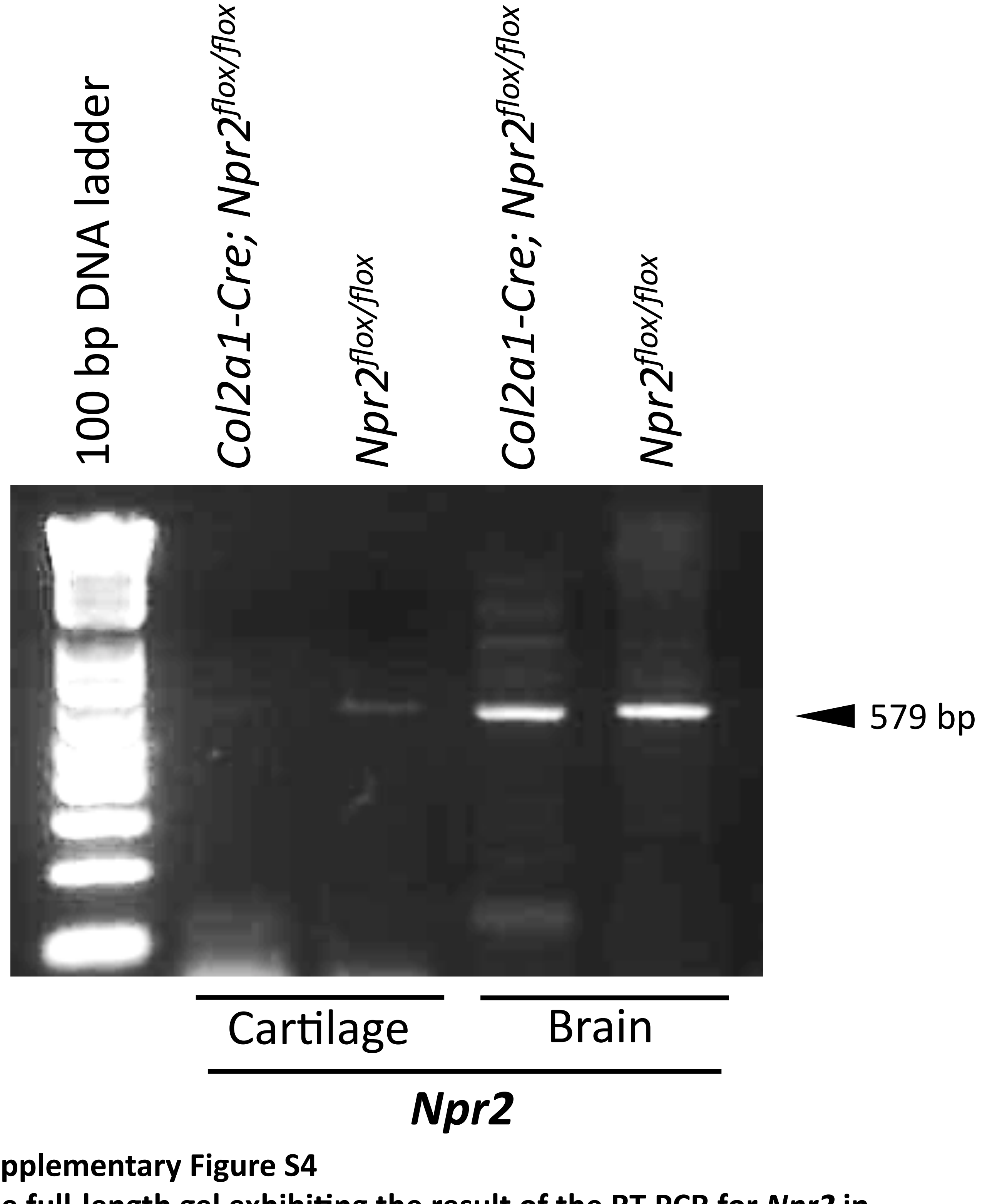


**Supplementary Figure S4**
**The full-length gel exhibiting the result of the RT-PCR for *Npr2* in cartilage and brain of *Col2-Cre; Npr2*$^{flox/flox}$ and *Npr2*$^{flox/flox}$ mice.**

Supplementary Figure

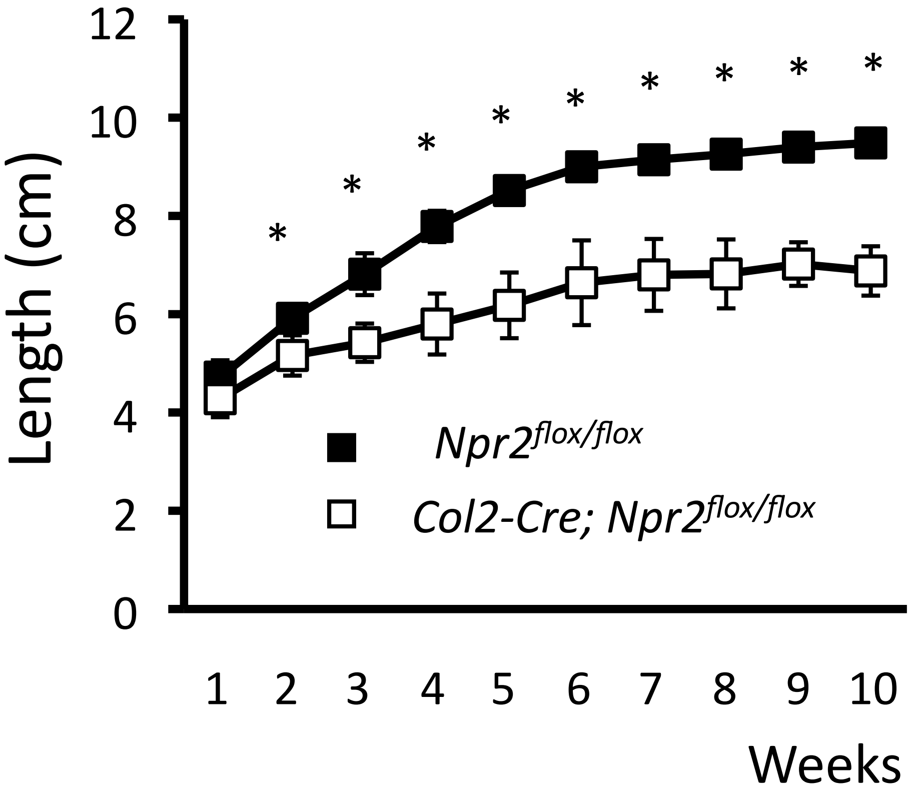


**Supplementary Figure S5**
**Growth curves of naso-anal length of *Npr2$^{flox/flox}$* (■) and *Col2-Cre; Npr2$^{flox/flox}$* (□) mice.**
*: P < 0.01.

Supplementary Figure

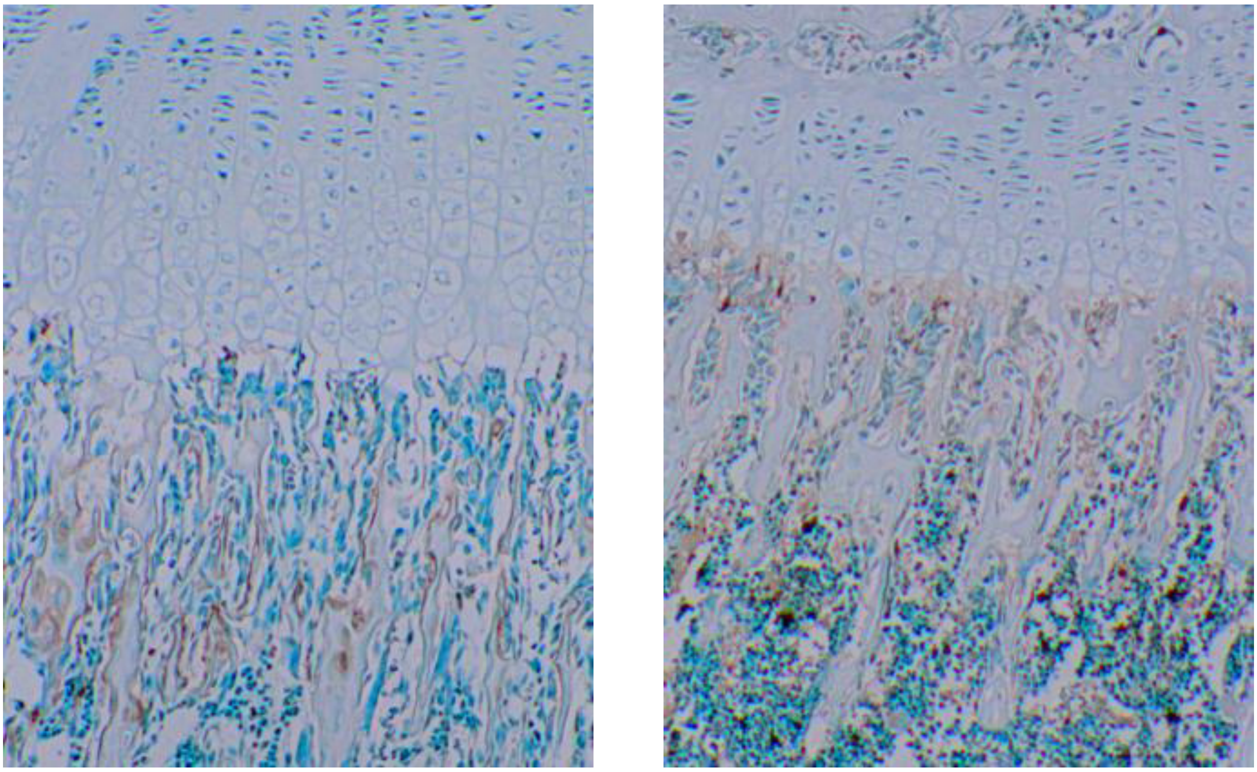

*Npr2*$^{flox/flox}$

*Col2a1-Cre; Npr2*$^{flox/flox}$

**Supplementary Figure S6**
**TUNEL staining of growth plates of *Npr2*$^{flox/flox}$ and *Col2-Cre; Npr2*$^{flox/flox}$ mice.**
